# The Effect of Recycling Flux on the Performance and Microbial Community Composition of a Biofilm Hydrolytic-Aerobic Recycling Process Treating Anthraquinone Reactive Dyes

**DOI:** 10.3390/molecules16129838

**Published:** 2011-11-25

**Authors:** Yuanpeng Wang, Kang Zhu, Yanmei Zheng, Haitao Wang, Guowen Dong, Ning He, Qingbiao Li

**Affiliations:** 1 Department of Chemical and Biochemical Engineering, College of Chemistry and Chemical Engineering, Xiamen University, Xiamen 361005, China; 2 State Key Lab of Urban Water Resource and Environment, Harbin Institute of Technology, Harbin 150001, China

**Keywords:** reactive blue 19, PCR-DGGE, microbial community structure, hydrolytic-aerobic recycling process, recycling flux

## Abstract

Synthetic dyes are extensively used and rarely degraded. Microbial decomposition is a cost-effective alternative to chemical and physical degradation processes. In this study, the decomposition of simulated anthraquinone reactive dye (Reactive Blue 19; RB19) at a concentration of 400-mg/L in wastewater by a biofilm hydrolytic-aerobic recycling system was investigated over a range of recycling fluxes. The 16S rDNA-based fingerprint technique was also used to investigate the microbial community composition. Results indicated that the recycling flux was a key factor that influenced RB19 degradation. The RB19 and COD removal efficiency could reach values as high as 82.1% and 95.4%, respectively, with a recycling flux of 10 mL/min. Molecular analysis indicated that some strains were similar to *Aeromonadales*, *Tolumonas*, and some uncultured clones were assumed to be potential decolorization bacteria. However, the microbial community composition in the reactors remained relatively stable at different recycling fluxes. This study provided insights on the decolorization capability and the population dynamics during the decolorization process of anthraquinone dye wastewater.

## 1. Introduction

Synthetic dyes are used extensively in textile dyeing, paper printing, color photography, cosmetics, and other industries. The amount of dyes produced worldwide is estimated to be 10,000 tons/year [1]. In addition to the unpleasant appearance of dye-polluted wastewater, most dyes and their potential breakdown products are toxic, carcinogenic, and mutagenic [2]. Anthraquinone dyes are one of the most commonly used types of synthetic dyes. The color of anthraquinone dyes is partially associated with the anthraquinone nucleus and is modified by the type, number, and position of the substituents. Because of the chemical stability of the anthraquinone nucleus, anthraquinone dyes degrade slowly [3,4]. A wide range of physical-chemical methods have been developed for removing anthraquinone dyes, such as adsorption on inorganic absorbents [5], UV/H_2_O_2_ oxidation [6], and chemical oxidation by ozone [7]. However, these methods are generally costly, and large volumes of toxic byproducts can be generated during the treatment process [8,9].

Mixed-culture biodegradation technologies, such as anaerobic, aerobic and anaerobic/aerobic processes, have been used to treat dye wastewater [10,11]. Anthraquinone dyes adsorb poorly onto biomass and cannot be readily degraded under the typical aerobic conditions for its high water solubility, so most research has been done on combined anaerobic/aerobic processes. The crucial step in the biodegradation of dyes is the removal of the chromogenic groups from the aromatic nucleus through either the reductive or hydrolytic enzymes of anaerobic microorganisms. Although most anaerobic microorganisms have the enzymes necessary for aromatic ring degradation, limitations of using anaerobic treatment have been observed. Fontenot *et al*. conducted laboratory experiments with a mixed methanogenic culture under an initial concentration of RB19 of more than 300 mg/L, which resulted in complete inhibition of methanogenesis [12]. For high concentration dye wastewater, VFA could be produced, which brings the wastewater into a low pH environment. If the pH is not controlled, the accumulation of VFA causes the wastewater to become seriously acidified, which deactivates the anaerobic microorganisms. Therefore, a combined hydrolytic-aerobic reactor recycling system was developed for the treatment of anthraquinone dye wastewater by Wang *et al*. [13]. This system consisted of a hydrolytic reactor operated under anoxic condition (DO 0–0.5 mg/L) and an aerobic biofilm reactor with aeration (DO 3–5 mg/L), which was operated via recirculation of the reactor contents. The removal efficiency of dye color and COD was improved significantly, and the reactors could be potentially used for the mineralization of anthraquinone dye in wastewater. The objectives of this study were to examine the effect of recycling flux on the degradation of anthraquinone dye in the hydrolytic-aerobic recycling process. Reactive Blue 19 was selected as a model anthraquinone dye for this study. The objectives were to compare color and COD removal efficiency in the recycling reactors under different recycling fluxes (5–15 mL/min) and to use the culture-independent molecular technique-denaturing gradient gel electrophoresis (DGGE) to characterize the changes of the microbial community structure in the reactors. This study may be valuable for the technological design of high concentration dye wastewater treatment by the hydrolytic–aerobic combination process.

## 2. Results

### 2.1. The Effect of Recycling Flux on Performance of RB19 Anthraquinone Dye Wastewater Degradation

**Figure 1:**
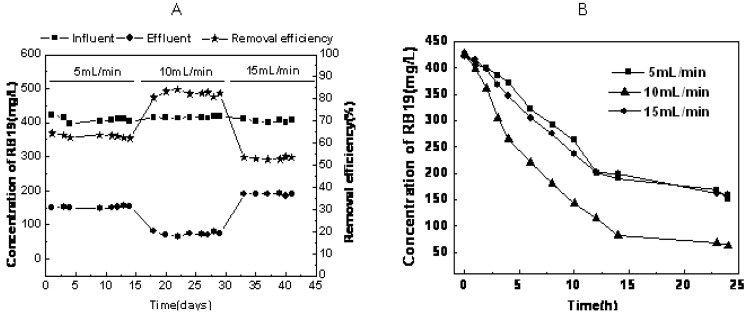
Time course of RB19 decolorization (**A**: during 42 days; **B**: during 24 h).

**Figure 2:**
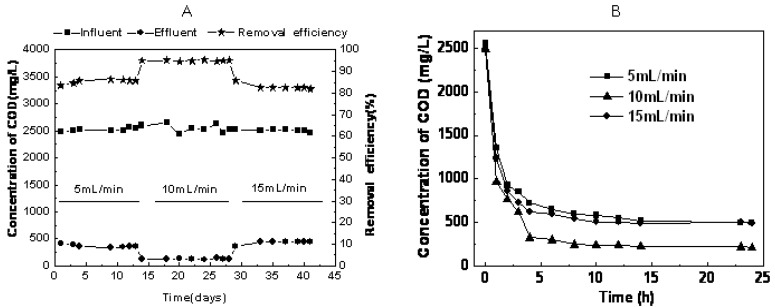
Time course of COD removal (**A**: during 42 days; **B**: during 24 h).

RB19 removal efficiency under different recycling flux regimes is shown in Figure 1A. When operating at a 5 mL/min and 10 mL/min recycling flux, RB19 removal efficiency could reach 63.0% and 85.4%, respectively, after 24 h degradation. The removal efficiencies dropped down to 53.2% when the recycling flux was further increased to 15 mL/min. For COD removal efficiency, the COD removal efficiency was 83.2% and 94.1% while operating with a recycling flux of 5 mL/min to 10 mL/min. The removal efficiency also dropped down to 82.5% when the recycling flux rate was increased to 15 mL/min (Figure 2A).

In addition, we can clearly observe from Figure 1B that the most effective decolorization took place in the first 12 h, but the rate of decolorization was fastest in the recycling reactor system operating at 10 mL/min. Figure 2B presents the changes of the COD removal rate with time for different recycling fluxes. The COD removal rate quickly decreased in the initial 4 h and then remained constant when the recycling rates were set at 5, 10 and 15 mL/min. The rate of COD removal was fastest in the recycling reactor system operating at 10 mL/min.

### 2.2. Microbial Community Composition during RB19 Wastewater Degradation

**Figure 3:**
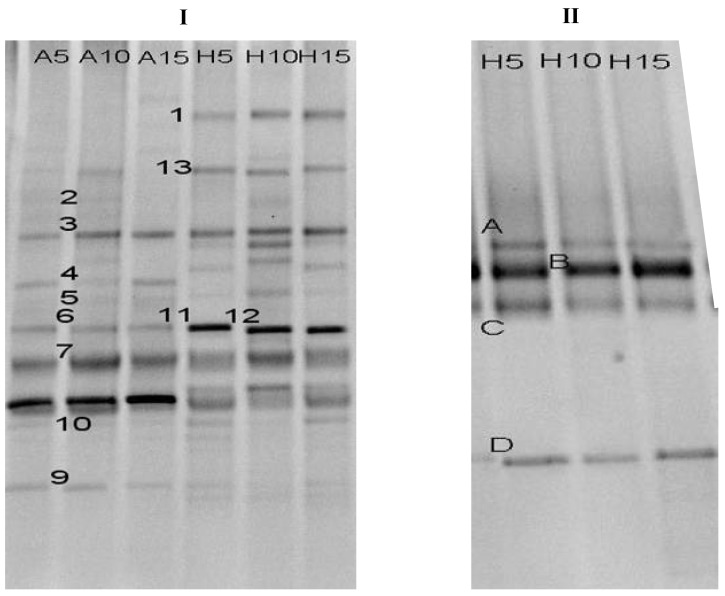
DGGE profile of bacteria (**I**) and archaea (**II**) in the biofilm hydrolytic-aerobic recycling systems at different recycling flux rates (A5, A10, A15: 5 mL/min, 10 mL/min, 15 mL/min from the aerobic reactor; H5, H 10, H 15: 5 mL/min, 10 mL/min, 15 mL/min from the hydrolytic reactor).

To further understand the anthraquinone degradation reactor and potentially improve decolorization performance, the microbial composition of the acclimatized RB19-degradation consortium was analyzed by PCR-DGGE. Figure 3 shows the successive changes in DGGE band profiles of the RB19-degradation consortium grown in aerobic and hydrolytic reactors (lane A5, A10 and A15 were from the aerobic reactor and lane H5, H10 and H15 were from the hydrolytic reactor) during the recycling flux tests. The dominant bands in the aerobic reactor and the hydrolytic reactor were quite distinct (Figure 3).

Although many bands were detected in all samples, no significant differences in the bacteria community with RB19 treatment were readily observed. As shown in Figure 3, the number of bands in the biofilm with RB19 did not change as compared to that of bulk biofilm at different recycling fluxes. In addition, there were four different archaea belonging to *Euryarchaeota* in the hydrolytic reactor: *Methanobacterium sp*. MB4; Uncultured *euryarchaeote*; Uncultured bacterium BHARS-HB-002; and *Methanospirillum* sp. Ki8-1. The results also indicated that the archaea community structures in the system were relatively stable in the hydrolytic reactor at varying recycling flux rates.

**Figure 4:**
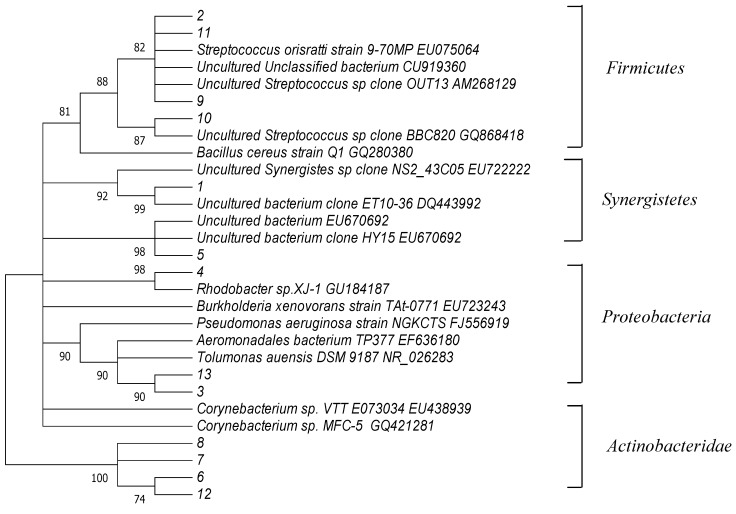
Phylogenetic tree of partial 16S rRNA gene sequence Bootstrap support values with 1,000 replicates are given along the branches.

### 2.3. Phylogenetic Analysis

A phylogenetic analysis of the sequences recovered from the DGGE gels was performed (Figure 4) where there were differences in the distribution of phylogenetic groups among the three processes. The closest matches of the obtained sequences to the known species were determined by comparison with the GeneBank database (Table 1). All of the sequences were closely matched to the known organisms previously recovered from environmental samples. A phylogenetic tree was generated with Mega.4.1. Sequences recovered from the DGGE gels were distributed throughout the tree. Based on this analysis, the recovered sequences were most closely matched to *Proteobacteria*, *Actinobacteria*, *Firmicutes*, and *Synergistetes*.

## 3. Discussion

In this study, higher removal efficiency of color and COD were achieved using the hydrolytic-aerobic recycling reactor system (Figure 1 and Figure 2). The crucial step in the biodegradation of dyes is the removal of chromogenic groups from the aromatic nucleus by either the reductive or hydrolytic enzyme activity of hydrolytic microorganisms. As shown in Table 1, band-3 was the closest to *Aeromonadales* bacterium TP377. According to Ren *et al*., *Aeromonas* could degrade anthraquinone dyes effectively [14]. Therefore, the microbe in the position of band-3 was assumed responsible for RB19 decomposition. Band-13 showed 95% sequence similarity to *Tolumonas auensis* DSM 9187 strain TA, which was detected from anoxic sediments of a freshwater lake and could degrade phenylalanine, phenylpyruvate, phenyllactate and phenylacetate to toluene [15]. It was suggested that this member might possess strong capabilities in degrading recalcitrant organic pollutants such as aromatic compounds under anaerobic conditions. Since toxic intermediates (e.g., aromatic amines) would be accumulated after anaerobic decolorization of anthraquinone dyes [16].

**Table 1:** Results of partial 16S rDNA sequences using BLAST in GenBank.

Band	Accession	Closely Related Sequence	Similarity
	No.		(%)
1	HM032045	Uncultured bacterium clone ET10-36(DQ443992)	99
2	HM032038	Uncultured bacterium (CU919360)	100
3	HM008699	*Aeromonadales* bacterium TP377 (EF636180)	95
4	HM032042	Uncultured *Streptococcus* sp clone BBC820	99
		(GQ868418)	
5	HM008698	Uncultured bacterium (EU670692)	99
6	HM032041	*Burkholderia xenovorans* strain	90
		TAt-0771(EU723243)	
7	HM032039	*Corynebacterium* sp. VTT E073034(EU438939)	98
8	HM032046	Corynebacterium sp. MFC-5(GQ421281)	98
9	HM032040	*Streptococcus orisratti* strain 9-70MP	100
		(EU075064)	
10	HM008700	*Rhodobacter* sp*.XJ-1* (GU184187)	90
11	HM008701	*Streptococcus* sp(AM268129)	93
12	HM032043	Uncultured *Synergistes* sp clone NS2_43C059	94
		(EU722222)	
13	HM032044	*Tolumonas auensis* DSM 9187 strain	95
		TA(NR_026283)	
A	HM008696	*Methanobacterium* sp. MB4 (DQ677518)	99
B	HM008697	Uncultured *euryarchaeote* (GU127414)	95
C	HM032047	Uncultured bacterium clone BHARS-HB-002	97
		(HM008697)	
D	HM032048	*Methanospirillum* sp. Ki8-1 (AB517986)	99

Although some bacteria related to RB19 degradation were found in the recycling reactors, the microbial community composition of the reactors experienced no significant change after increasing the recycling flux 5 mL/min to 15 mL/min (Figure 3). When the recycling flux increased from 5 mL/min, 10 mL/min and finally to 15 mL/min, the cycle time of the hydrolytic-aerobic reactors reduced from 10 h, 5 h and 3.3 h, respectively (Table 2). In previous research, some results demonstrated that retention time could serve as a selective factor for bacterial community development [17]. However, Schneider *et al*. proved that all major community components were present in reactors at different retention times [18]. Therefore, in this study, the reason for the difference between RB19 and COD removal efficiencies at different recycling flux was the different distribution of the cycle time in the hydrolytic and aerobic reactor not the shift of the microbial community structure in the system.

**Table 2:** Parameters of different operational periods.

Operational conditions ^a^	1	2	3
Hydraulic residence time (h)	24	24	24
Glucose (mg/L)	2000	2000	2000
RB19 feed (mg/L)	400	400	400
Recycling flux (mL/min)	5	10	15
Hours per cycle *	10	5	3.3
Cycles per phase **	2.4	4.8	7.3
Days per phase ***	14	14	14

^a^ Established during the hydrolytic-aerobic recycling process. * The time that the solution in the hydrolytic reactor was circulated into the aerobic reactor by one peristaltic pump, which is equal to the time that the solution in the aerobic reactor was circulated into the hydrolytic reactor at the same recycling flux. ** The number of times the solution was recycled between the hydrolytic reactor and the aerobic reactor within 24 h. *** The number of operation days of the hydrolytic-aerobic recycling process conducted at one recycling rate.

The removal of chromogenic groups is a rate-limiting step in the degradation of anthraquinone dyes [19], and the degradation efficiency of anthraquinone dyes depends mainly on the microbial capacity to remove these chromogenic groups. In the hydrolytic-aerobic recycling process, hydrolytic microorganisms in the hydrolytic reactor carried out the initial reductive decolorization step. Increased recycling flux enhances the solution circulating between the hydrolytic reactor and the aerobic reactor. The intermediate products of decolorization in the hydrolytic reactor could thus be timely decomposed by aerobic microorganisms [20]. Therefore, the metabolic and kinetic limitations to anaerobic microorganisms could be overcome in the recycling process. Therefore, RB19 removal efficiency and rate could effectively be improved when solutions recycle between the hydrolytic reactor and the aerobic reactor.

However, when the recycling rate exceeded a certain value, there was not enough time to insure that chromogenic groups could be completely removed from the dye in the hydrolytic reactor, leading to reduced RB19 removal efficiency and rate within the same degradation time [12]. When the recycling flux is increased from 10 mL/min to 15 mL/min, the cycle time of the hydrolytic reactor reduced from 5 h to 3.3 h. Therefore, the chromogenic groups from the aromatic nucleus were transferred to the aerobic reactor without being removed completely in the hydrolytic reactor, causing a decline in the RB19 removal efficiency. Similarly, the cycle time of intermediates generated in the hydrolytic reactor was too short to be oxidized in the aerobic bioreactor, leading to a declining RB19 removal rate. Therefore, the recycling flux was too high to improve the COD and RB19 removal rate. Considering the optimal decolorization efficiency, an optimal recycling flux rate of 10 mL/min was determined to achieve the highest decolorization efficiency in this study.

## 4. Experimental

### 4.1. Dye and Reagent

**Figure 5:**
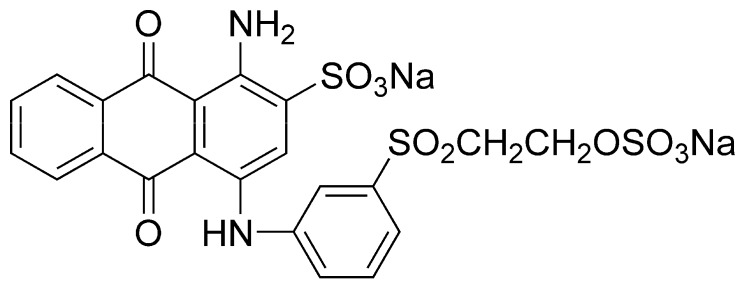
Chemical structure of Reactive Blue 19.

The commercial anthraquinone dye, C.I. Reactive Blue 19 (Figure 5) used in this study was provided by the Xiamen Hualun Group (Xiamen, China) and used without further purification. All other chemicals were of analytical grade.

### 4.2. Reactors, Microorganisms and Synthetic Wastewater

The hydrolytic and aerobic microorganisms were obtained from activated sludge provided by the Xiamen Domestic Sewage Treatment Plant. Hydrolytic and aerobic microorganisms were simultaneously cultured in the two sets of reactors. Both reactors were filled with D-2 soft fiber that was 30 cm in height. The exact physical characteristics of the biofilm are shown in Table 3. During the acclimation period, to improve the microorganisms’ adaptability to the Reactive Blue 19 wastewater, the Reactive Blue 19 concentration was increased stepwise from 0 to 400 mg/L in approximately 1 month with 2,000 mg/L of glucose as an external carbon source. The composition of the synthetic dye wastewater in the assay contained the following components (g/L): RB19 (from 0 to 0.4), glucose 2.0, NaHCO_3_ 1.5, NH_4_Cl 0.07, and KH_2_PO_4_ 0.03. The assay contained the following concentration of trace elements (mg/L): CaCl_2_·6H_2_O 0.01, FeSO_4_·7H_2_O 1.55, MnSO_4_ 4.95, ZnSO_4_·7H_2_O 0.71, CuSO_4_·5H_2_O 0.48, and CoCl_2_·6H_2_O 0.01.

**Table 3:** Principal physical characteristics of the biofilm hydrolytic and aerobic reactors.

Physical characteristics	Hydrolytic reactor	Aerobic reactor
Total volume of reactor (L)	4.5	4.5
Working volume of reactor (L)	3	3
Number of soft fiber carrier	16	16
Soft fiber carrier density (g/cm3)	0.91	0.91
Soft fiber carrier height (m)	0.3	0.3
Packing dry weight (g)	0.624	0.624
Specific surface areas of the carrier(m^2^/m^3^)	5.56	5.56
Total surface areas of the carrier (m^2^)	3.81	3.81

### 4.3. Experimental Procedure

For comparison, all experiments were conducted at 400 mg/L RB19 and 2,000 mg/L glucose (COD 2,100 mg/L). Before each run, the simulated wastewater was prepared by adding RB19 and glucose into tap water. The pH of the simulated wastewater was adjusted to approximately 7.5 using a NaOH and HCl solution.

**Figure 6:**
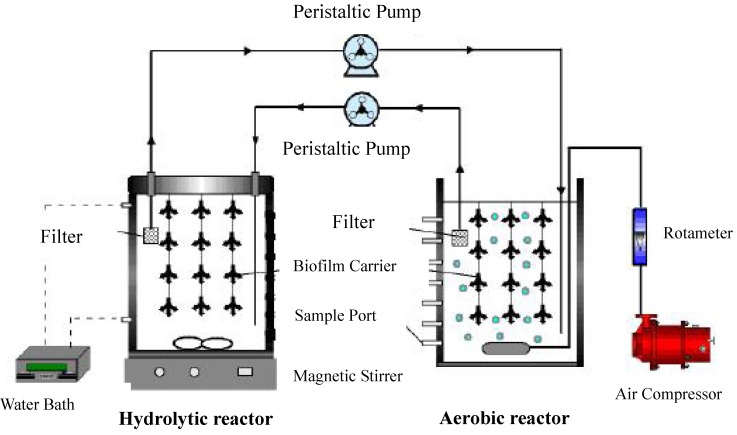
Bench-scale biofilm hydrolytic-aerobic recycling process.

The bench-scale biofilm hydrolytic-aerobic recycling process is presented in Figure 6. The hydrolytic reactor was covered with a plastic cover unsealed where there were two holes to allow the tubes to insert. The hydrolytic reactor was maintained at 35 ± 1 °C, and the solution in it was mixed by a magnetic stirrer, which doubled as a heater. The solution in the aerobic reactor was aerated using an air compressor with an aeration rate of 3 L/min at room temperature (20–25 °C), and the DO (dissolved oxygen) was greater than 4 mg/L. The hydrolytic-aerobic recycling process was performed by means of two external recirculation peristaltic pumps (BT01-100) in continuous recirculation 24 h at the specified recycling flux (5, 10, and 15 mL/min). The simulated wastewater was simultaneously added to the hydrolytic and aerobic reactor. Then, the two peristaltic pumps were activated. The effluent of the hydrolytic reactor was circulated into the inlet of the aerobic reactor by the peristaltic pump that drew liquid though the filter from the top of the hydrolytic reactor and transferred it to the bottom of the aerobic reactor to ensure good internal mixing of the liquid in the reactor. Meanwhile, the effluent of the aerobic reactor was circulated into the inlet of the hydrolytic reactor by a peristaltic pump that drew liquid though the filter from the top of the aerobic reactor and transferred it to the bottom of the aerobic reactor. The operation conditions for both reactors are listed in Table 2.

The main parameters such as COD, pH and RB19 concentration were measured after centrifugation of the samples collected at the sample ports of the hydrolytic and aerobic reactor at different time intervals. The samples were kept at 4 °C in a freezer before analysis.

To analyze the microbial community dynamic in the biofilm hydrolytic-aerobic recycling system treating RB19, the batch decolorization experiments were performed at different recycling fluxes. When the removal efficiency became stable under each recycling flux, the microorganisms were collected from the biofilm of the combined aerobic-hydrolytic reactor system, placed on sterile polypropylene tables, and stored at −20 °C before DNA extraction.

### 4.4. Calculation Method in the Combined Reactor System

The total removal efficiency of COD (*R*tc) and that of RB19 (*R*tr) during the biodegradation process was calculated according to the following equation [13]:

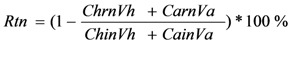
(1)
where, *n* is COD or RB19; *C*ain is the initial concentration of *n* in the aerobic reactor (mg/L); *Chin* is the initial concentration of *n* in the hydrolytic reactor (mg/L); *C*arn is the residual concentration of *n* in the aerobic reactor after degradation of 24 h (mg/L); *C*hrn is the residual concentration of *n* in the hydrolytic reactor after degradation of 24 h (mg/L); *V*a is the effective volume of the aerobic reactor (L); and *V*h is the effective volume of the hydrolytic reactor (L).

### 4.5. PCR-DGGE

Approximately 1 g of the microorganisms was weighed into DNA extraction tubes using the 3S DNA Isolation Kit V2.2 (Shenery Biocolor). PCR amplification for DGGE analysis, details of the DGGE protocol, gel staining, band visualization, and pattern analysis have been described elsewhere [21]. Briefly, PCR amplicons were generated using the generally conserved 16S rDNA gene primer pair F338 and R518 for the amplification of bacterial 16S rDNA genes [21]. The primer pair ARC334 and ARC915 was selected for the amplification of archaea 16S rDNA genes [22]. The GC-clamp described by Muyzer *et al*. was added to the forward primers to facilitate the DGGE [23]. For DGGE analysis, 400 ng of PCR product generated from each sample was separated on an 8% acrylamide gel with a linear denaturant gradient range of 35 to 60% using the Bio-Rad D-GENE System. Gels were stained with SYBR Green I and bands were visualized using a Bio-Rad Gel Doc 1000 and Molecular Analyst software (Bio-Rad Laboratories).

### 4.6. Phylogenetic Analysis

The sequences recovered from excised bands were compared with other sequences previously deposited in GenBank, using the Means Basic Local Alignment Search Tool (BLAST) [24]. Sequence alignment and phylogenetic analysis was performed using Mega 4.1 software. The phylogenetic tree was constructed in Mega using the neighbor-joining method.

### 4.7. Nucleotide Sequence Accession Numbers

The nucleotide sequences determined in this study have been deposited in the GenBank database under the accession numbers HM032038-HM032048 and HM008696-HM008701.

## 5. Conclusions

The DGGE of PCR-amplified 16S rDNA genes were used to analyze the bacterial community dynamics in a biofilm hydrolytic-aerobic recycling system treating an anthraquinone dye. Firstly, the different operation conditions in the aerobic and hydrolytic reactors determined the discrepancy between the microbial communities in the two reactors. Secondly, The RB19 and COD removal efficiency with a concentration of RB19 at 400 mg/L could reach up to 82.1% and 95.4% at a recycling flux of 10 mL/min, respectively. The 16S rDNA-based molecular analysis techniques demonstrated that some strains similar to *Aeromonadales*, *Tolumonas*, and some uncultured clones acted for the dynamic succession. Finally, the bacterial community structures in the system were relatively stable, and the different distribution of HRT in the hydrolytic and aerobic reactors resulted in the difference between RB19 and COD removal efficiencies at varying recycling fluxes. From the above results, this study provided insights on the decolorization capability and the population dynamics during the decolorization process of anthraquinone dye wastewater.
